# Glycine and Melatonin Improve Preimplantation Development of Porcine Oocytes Vitrified at the Germinal Vesicle Stage

**DOI:** 10.3389/fcell.2022.856486

**Published:** 2022-02-24

**Authors:** Yu Tang, Ying Zhang, Lixiang Liu, Yifeng Yang, Yan Wang, Baozeng Xu

**Affiliations:** ^1^ Institute of Special Animal and Plant Sciences, Chinese Academy of Agricultural Sciences, Changchu, China; ^2^ State Key Laboratory for Molecular Biology of Economic Animals, Chinese Academy of Agricultural Sciences, Changchun, China

**Keywords:** porcine oocyte, vitrification and thawing, *in vitro* maturation and fertilization, embryonic development, osmotic stress, oxidative stress, glycine, melatonin

## Abstract

Lipid-rich porcine oocytes are extremely sensitive to cryopreservation compared to other low-lipid oocytes. Vitrification has outperformed slowing freezing in oocyte cryopreservation and is expected to improve further by minimizing cellular osmotic and/or oxidative stresses. In this study, we compared the effects of loading porcine cumulus-oocyte complexes with glycine (an organic osmolyte) or glycine plus melatonin (an endogenous antioxidant) during vitrification, thawing and subsequent maturation to mitigate osmotic injuries or osmotic and oxidative damages on the developmental potential of porcine oocytes. Our data demonstrated that glycine treatment significantly increased the vitrification efficiency of porcine oocytes to levels comparable to those observed with glycine plus melatonin treatment. It was manifested as the thawed oocyte viability, oocyte nuclear maturation, contents of reactive oxygen species, translocation of cortical granules and apoptotic occurrence in mature oocytes, levels of ATP and transcripts of glycolytic genes in cumulus cells (markers of oocyte quality), oocyte fertilization and blastocyst development. However, the latter was more likely than the former to increase ATP contents and normal mitochondrial distribution in mature oocytes. Taken together, our results suggest that mitigating osmotic and oxidative stresses induced by vitrification and thawing can further enhance the developmental competency of vitrified porcine oocytes at the germinal vesicle stage.

## Introduction

Cryopreservation is widely used to preserve structurally intact living oocytes of mammals for the widespread and long-term storage of animal genetic resources in very low temperatures, typically in liquid nitrogen at −196°C. The great differences in sensitivity to chilling and freezing injuries depend on different lipid contents in oocytes (Arav, 2014; Quan et al., 2017). In comparison with oocytes of mouse, bovine, or sheep, porcine oocytes have the highest lipid content and are most sensitive to chilling and cryopreservation (Loewenstein and Cohen, 1964; Isachenko et al., 1998; McEvoy et al., 2000). Porcine oocytes cryopreserved at the germinal vesicle (GV) stage have the potential to undergo nuclear maturation, fertilization, and thus produce viable piglets following embryo transfer (Somfai et al., 2014). However, cryopreserved oocytes still yield very low blastocysts compared to fresh oocytes (Casillas et al., 2018). This implies that cryopreservation may cause a certain degree of sublethal damage to porcine or other lipid-rich oocytes, thus further optimization is needed to enhance the cryopreservation efficiency of lipid-rich oocytes.

Cryodamage that compromises oocyte quality can be the result of multiple factors, including ice nucleation, osmotic stress, and oxidative injury. Although either slow freezing or vitrification techniques for oocyte cryopreservation are widely used, there is growing evidence that vitrification is more efficient for mammalian oocytes preservation than slow freezing (Rho et al., 2002; Chen et al., 2003; Sanchez-Partida et al., 2011). Compared to slowing freezing cryopreservation, vitrification requires much greater concentrations of cryoprotectants to eliminate mechanical damage caused by ice, bypass the steps of finding the optimal cooling and warming rates, transcend the need for specialized equipment to regulate the cooling rate, and render cooling/warming to be fast enough to outpace chilling damage, but it introduces greater cryoprotectant toxicity and risk of osmotic stress during the addition and removal of cryoprotectants, which are detrimental to cell viability (Kuleshova and Lopata, 2002; Mullen et al., 2004). Moreover, accumulating evidence demonstrates that oxidative stress is another adverse factor jeopardizing the developmental potential of cryopreserved gametes (Somfai et al., 2007; Gupta et al., 2010; Tatone et al., 2010; Gualtieri et al., 2021). Vitrification seriously affects the morphological and functional integrity of oocytes’ mitochondria and endogenous antioxidant system, resulting in elevated levels of reactive oxygen species (ROS) (Gupta et al., 2010; Zhao et al., 2011). Excessive ROS, in turn, can cause mitochondrial damage, ATP depletion, meiotic spindle disassembly, apoptosis and compromised developmental capacity in oocytes and early embryos (Choi et al., 2007; Liang et al., 2016; Maldonado et al., 2016).

Osmolarity is intimately connected with cell volume regulation, which is implicated as a key factor affecting oocyte and embryo development. The *in vitro* culture of mammalian preimplantation embryos has had over 100 years history, but it was not until the late 1980s and early 1990s that a critical breakthrough was achieved with the medium that allowed fertilized oocytes from female mice to develop *in vitro* to the blastocyst stage (Biggers, 1998). The major difference is that the osmolarity of the successful embryo culture media is significantly lower than previous culture media. The osmolarities of successful media, such as KSOM and CZB, are in the range of 250–275 mOsM, which is much lower than that of the physiological oviduct fluid (300–310 mOsM) (Baltz and Tartia, 2010). On the other hand, preimplantation embryos would develop at higher osmolarities *in vitro* if any of several compounds such as glycine, betaine or glutamine were present in the culture media (Dawson and Baltz, 1997). This is because these compounds in the embryo act as organic osmolytes, a class of diverse, small, neutral organic compounds accumulated by cells to provide intracellular osmotic support in place of ions that can interrupt cellular physiology at higher concentrations (Yancey et al., 1982; Zhou and Baltz, 2013). Among them, glycine appears to have the highest level of protection against hypertonic media (Van Winkle et al., 1990; Hadi et al., 2005). Moreover, the fully grown GV oocytes of the mouse can accumulate glycine via the GLYT1 transporter to control the cell volume when ovulation is triggered *in vivo* or oocytes are removed from follicles and cultured *in vitro* (Tartia et al., 2009). Parallelly, our previous study suggests that reducing osmotic stress induced by vitrification/thawing using glycine supplementation could improve the development of vitrified mouse oocytes (Cao et al., 2016). However, whether the mechanism of cell volume regulation with glycine is shared between porcine and mouse oocytes and preimplantation embryos remains unclear. Therefore, it is necessary to clarify the impacts of adding glycine to reduce osmotic damage due to drastic changes in cell volume or intracellular osmolytes’ concentration and species during the vitrification/thawing of porcine oocytes.

There is increasing evidence that oxidative and osmotic injuries are closely interrelated. Osmotic stress stimulates the generation of superoxide anion in mammalian somatic and gametic cells (Lambert, 2003; Ortenblad et al., 2003; Lambert et al., 2006; Burnaugh et al., 2010), and vice versa, at least in part (Schliess et al., 2006; Rosas-Rodriguez and Valenzuela-Soto, 2010). However, the pathways that regulate the cellular response to oxidative and osmotic stress in mammalian oocytes, especially during vitrification, are not well understood. We hypothesize that osmotic stress and oxidative stress interact in a mutually amplifying loop during vitrification/thawing of oocytes, reducing osmotic stress with organic osmolytes or mitigating osmotic and oxidative stresses with physiological antioxidant and organic osmolytes together could enhance the developmental competency of vitrified porcine oocytes comparably. Melatonin (MT), an indoleamine synthesized and secreted by the pineal gland, acts as a potent free radical scavenger and a natural broad-spectrum antioxidant (Barlow-Walden et al., 1995; Reiter et al., 2002). Melatonin addition has been demonstrated to promote the efficiency of vitrified oocytes and embryos in various species (Mehaisen et al., 2015; Zhang et al., 2016; Zhao et al., 2016). To our knowledge, no prospective study has been reported on the supplementation of glycine and melatonin during porcine oocyte vitrification. The present study was aimed to compare the effects between lessening osmotic stress with glycine supplementation and cutting down osmotic and oxidative stresses with glycine and melatonin supplementation on the developmental competency of porcine oocytes vitrified at the germinal vesicle (GV) stage during vitrification, thawing and followed maturation, as determined by (1). Survivability of oocytes undergone vitrification, thawing and *in vitro* maturation and the ability of the vitrified oocyte to progress to the blastocyst stage following *in vitro* oocyte maturation and fertilization, (2). ROS levels in oocytes at the second metaphase stage (MII oocytes), (3). Translocation of cortical granules during oocyte meiosis, (4). Rate of apoptosis in MII oocytes, (5). Fluorescent intensity and distribution pattern of functional mitochondria, (6). Levels of ATP and transcripts of glycolytic genes in cumulus cells attached to MII oocytes, and (7). ATP contents in MII oocytes.

## Materials and Methods

### Reagents and Antibodies

All reagents and chemicals used in this study were from Sigma Chemical Company (St. Louis, MO, United States) unless otherwise stated. MitoTracker Red CMXRos was purchased from Thermo Fisher (Catalog# M7512); Annexin-V staining kit was obtained from Vazyme (Catalog# A211-01); Lens Culinaris Agglutinin (LCA)-FITC was purchased from Thermo Fisher (Catalog# l32475); Reactive Oxygen Species Assay Kit was produced by Beyotime (China, Catlog# SOO33S).

### Collection of Porcine Oocytes

Porcine ovaries obtained from prepubertal gilts at a local slaughterhouse were preserved in saline supplemented with 100 units/ml penicillin G and 0.1 mg/ml streptomycin sulfate and delivered to the laboratory within 2 h at 35°C. Fully-grown oocytes wrapped by cumulus cells at the germinal vesicle (GV) stage, called cumulus-oocyte complexes (COCs), were aspirated from antral follicles (3–7 mm in diameter) using an 18-gauge needle attached to a 20 ml disposable syringe. Oocytes with three or more intact cumulus cell layers and uniform granulated cytoplasm, without apparent signs of lysis, were used for experiments.

### Vitrification and Warming of Porcine Oocytes at the GV Stage

Porcine COCs were vitrified by the cryoleaf (Cooper Surgical Company, Origio, USA) method as previously described (Somfai et al., 2014) with minor changes. Briefly, the COCs were initially incubated in the basic medium (BM), which was composed of modified NCSU-37 (Ocampo et al., 1993) without glucose, 20 mM HEPES, 50 mM β-mercaptoethanol, 0.17 mM sodium pyruvate, 2.73 mM sodium lactate, 4 mg/ml bovine serum albumin (BSA) and 7.5 mg/ml cytochalasin B, for 30 min. The COCs were transferred into an equilibration medium (EM) for 13–15 min. The EM was BM supplemented with 2% (v/v) ethylene glycol (EG) + 2% (v/v) propylene glycol (PG). The abovementioned incubation in the BM and EM was completed at 38.5°C in an atmosphere of 5% CO _2_ and saturated humidity. After equilibration, a group of 25–30 COCs were loaded into the Rapid-i hole (arc carrier) with 2–3 µl of vitrification solution (BM supplemented with 50 mg/ml polyvinylpyrrolidone (PVP), 0.3 M sucrose, 17.5% (v/v) EG, and 17.5% (v/v) PG) following three washes in 20 µl vitrification solution. The Rapid-i was placed in liquid nitrogen (LN) vapor for 30 s and inserted into a pre-cooled RapidStraw (a hollow cylindrical tube). The RapidStraw was sealed at the top and then submerged into LN. Efficient vitrification could be achieved if the process can be completed in 30–40 s or less from the time the oocytes were transferred to the vitrification medium until the completion of vitrification. For the warming of vitrified COCs, the Rapid-i with COCs was moved from LN and plunged into 1,000 µl pre-warmed warming solution (0.4 M sucrose in BM) for 1–2 min. The warmed COCs were sequentially incubated in 500 µl BM supplemented with 0.2, 0.1, 0.05, and 0 M sucrose for 1 min each, and then thoroughly washed in the BM. The whole thawing procedure was performed at 38.5°C.

### *In Vitro* Maturation, Fertilization and Preimplantation Embryo Culture

For *in vitro* maturation (IVM) of oocytes, every 50 thawed COCs was sequentially cultured in 500 μl MI- and MII-medium in four-well culture plates (Nunc, Roskilde, Denmark) at 38.5°C in an atmosphere with 5% CO _2_ and saturated humidity for 22 h each. MI medium was improved TCM-199 (Gibco, Grand Island, NY, United States) containing 50 ug/ml streptomycin sulfate, 75 ug/ml potassium penicillin G, 10% porcine follicular fluid (PFF), 10 IU/ml Luteinizing Hormone (LH), 10 ng/ml epidermal growth factor (EGF), 10 IU/ml Follicle Stimulating Hormone (FSH), and 100 IU/ml Insulin Transferrin Selenium (ITS). While MII-medium was MI-medium without LH and FSH supplementations.

For oocyte fertilization *in vitro* (IVF)*,* oocytes were denuded by repeated pipetting in the maturation medium supplemented with 300 IU/ml hyaluronidase at the end of IVM. A group of 15 cumulus-free oocytes with visible first polar bodies (MII oocytes) were washed three times in modified Tris Buffered Medium (mTBM, the fertilization medium) and transferred into a 60 µl mTBM droplet covered with paraffin oil at 38.5°C in an atmosphere of 5% CO_2_ for IVF. The mTBM was consist of (in mM) 113.1 NaCl, 7.5 CaCl_2·_2H_2_O, 3.0 KCl, 11.0 glucose, 20.0 Tris Base, 5.0 Na-pyruvate, and 1.0 caffeine and 0.2% BSA. The frozen porcine sperms stored in a sperm capillary were thawed in a 37°C water bath for 1 min, then washed twice in mTBM by centrifugation at 700 *g* for 3 min. These centrifugated sperms were resuspended with 1 ml pre-warmed mTBM and incubated at 38.5°Cfor 15 min. MII oocytes were inseminated in mTBM medium for 6 h with 1 × 10^5^ swim-up sperms/ml covered with paraffin oil at 38.5°C in an atmosphere of 5% CO_2_ and saturated humidity. After removing sperms from the surface of the zona pellucida by repeated pipetting in porcine zygote medium-3 (PZM-3) (Yoshioka et al., 2002), a group of 10 presumable zygotes was cultured in 30 µl drops of PZM-3 in 35-mm plastic dishes under paraffin oil at 38.5°C in an atmosphere of 5% CO_2_ and saturated humidity. The percentages of embryonic cleavage and blastocyst were recorded on day 2 and 7, respectively.

### Oocyte Viability Assay After Maturation Culture

The post-maturation viability of oocytes was judged by their morphological characteristics. Oocytes were considered as the living oocytes if their shape was spherical, their cytoplasm was uniformly granulated with refraction, and there was an obvious perivitelline space between the zona pellucida and the cytoplasmic membrane. Oocytes were judged as the dying or dead oocytes if their shape is abnormal, their cytoplasm is dim and non-refracted, or there was no obvious gap between the zona pellucida and the cytoplasmic membrane.

### Evaluation of Oocyte Nuclear Maturation

The denuded oocytes were fixed with 4% paraformaldehyde (PFA) in PBS for 30 min at room temperature (fixation). Then, oocytes were transferred into permeabilizing solution (PBS supplemented with 2% Triton X-100) at 37°C overnight (permeabilization). After incubation in the blocking buffer (3% BSA in PBS) for 1 h at room temperature (blocking), oocytes were incubated with Rhodamine phalloidin (Invitrogen, Cat# R415) diluted 1:50 in the blocking buffer for 40 min at room temperature. After washing thoroughly in the washing buffer (PBS supplemented with 0.1% Tween-20, 0.01% Triton X-100), the oocytes were transferred to a small drop of Prolong Antifade mounting medium containing 4, 6-diamidino-2-phenylindole (DAPI) (Invitrogen, Cat# P36962) and mounted on microscope slides. Observed under a Nikon laser scanning confocal microscope (Nikon C2 plus Si), only if the oocyte contained two clusters of chromosomes within the zona pellucida, one of which was surrounded by one or two small rings of microfilaments and the other was surrounded by a large ring of microfilament, such oocyte was considered as the MII oocyte.

### ROS Detection and Quantitation

ROS production in oocytes was determined using the Reactive Oxygen Species Assay Kit (ROS Assay Kit; Beyotime, China, Cat# SOO33S) according to the manufacturer’s instructions. Briefly, denuded oocytes were incubated in TCM-199 medium supplemented with 0.1% PVA and 10 μM DCFH diacetate (DCFHDA) at 38.5°C in a 5% CO_2_ incubator for 30 min. These oocytes were washed 3 times in DPBS containing 0.1% PVA, then placed on a glass slide and imaged with a confocal laser scanning microscope (Nikon C2, Japan) within 5 min under the same parameters. The fluorescence intensity of ROS was quantified using ImageJ 1.4 (National Institutes of Health, Bethesda, MD) and expressed in arbitrary fluorescence intensity units (A.U.).

### Distribution of Cortical Granules

The zona pellucida of oocytes was removed by treatment with 1% HCl in PBS at room temperature for 20 s. After fixation, permeabilization and blocking as mentioned above, the zona pellucida-free oocytes were incubated with FITC-Lens Culinaris Agglutinin (LCA) (100 μg/ml; ThermoFisher) in a blocking buffer for 30 min in dark. After three washes for 5 min each, oocytes were mounted on glass slides with Prolong Antifade mounting medium containing DAPI and examined with a Nikon laser scanning confocal microscope (Nikon C2 plus Si).

### Assessment of Early Apoptosis

Early apoptosis of oocytes was evaluated using Annexin V FLUOS Staining Kit (Roche, Germany) as previously described (Cao et al., 2016). Briefly, the cumulus-free oocytes were incubated in a liquid mixture containing Annexin-V fluos, Annexin-V buffer and PI for 30 min at room temperature in a dark box. After thorough washing with the washing buffer, oocytes were mounted on glass slides with their nuclear status evaluated by staining with the Prolong Antifade mounting medium with DAPI and imaged with a confocal laser scanning microscope (Nikon C2, Japan) under the same parameters within 1 week. The fluorescence intensity of each dye was quantitated using ImageJ 1.4 (National Institutes of Health, Bethesda, MD) and expressed in arbitrary fluorescence intensity units (A.U.). The oocytes were divided into three categories based on the results of immunofluorescence staining as previously described (Anguita et al., 2009): 1) Non-apoptotic oocyte: oocyte without Annexin-V staining in its cytoplasmic membrane; 2) Early apoptotic oocyte: The cytoplasmic membrane of oocyte specifically bound to Annexin-V, making the cytoplasmic membrane green; and 3) Necrotic oocyte: PI passed through the cytoplasmic membrane of the oocyte to stain the oocyte nucleus red.

### Mitochondrial Labeling and Measurement of Fluorescence Intensity

Mitotracker Red (Mitotracker Red FM; Molecular Probes, Eugene, OR, United States) was used to label the functional mitochondria according to the manufacturer’s instructions. Briefly, cumulus-free oocytes were incubated in M199 containing 0.1% PVA and 500 nM Mitotracker Red for 30 min at 38.5°C in an atmosphere of 5% CO_2_ and saturated humidity. After three washes of 5 min each in M199 containing 0.1% PVA, oocytes were mounted on glass slides with their nuclear status evaluated by staining with the Prolong Antifade mounting medium with DAPI following oocyte fixation. Oocytes were imaged with a confocal laser scanning microscope (Nikon C2, Japan) under the same parameters within 1 week. The fluorescence intensity of mitochondrion was quantitated using ImageJ 1.4 (National Institutes of Health, Bethesda, MD) to draw a perimeter enclosing each oocyte and determining the average intensity within the enclosed area, expressed in arbitrary fluorescence intensity units (A.U.).

### Quantitative Real-Time PCR

Relative transcript levels of genes involved in glycolysis in cumulus cells (CCs) were determined as we previously described (Fan et al., 2021). Briefly, RNA was extracted from an independent set of CCs derived from 30 COCs using the RNeasy Mini Kit (Qiagen, Hilden, Germany) according to the manufacturer’s instructions. RNA concentrations were determined using a Denovix DS-11 + spectrophotometer (DeNovix Inc., Wilmington, Delaware, USA). RNA (1 μg) from each sample was reverse-transcribed using the RevertAid First Strand cDNA Synthesis Kit (Thermo Scientific) and random hexamers. The amount of cDNA in each sample after reverse transcription was quantified by quantitative PCR following the standard protocol of the Light Cycler 480 SYBR Green I Master (Roche Applied Science, Penzberg, Germany) kit, which started with at 95°C for 10 min, followed by 40 cycles (denaturation for 30 s at 95°C, annealing and extension at 60°C for 30 s). The primer pairs used in this study are listed in Table 1. The specificity of each qRT-PCR amplicon was verified by melting curve analysis and DNA sequencing at Sangon Biotech Company (Shanghai, China). In each qRT-PCR run, a “template-free control” was applied to explore the presence of primer-dimers or contamination. The *GAPDH* gene was used as a housekeeping gene. Relative changes in the abundance of mRNA transcripts in different samples were determined using the 2^−ΔΔCT^ method (Livak and Schmittgen, 2001).

**TABLE 1:** Primers used for RT-PCR analyses.

Gene symbol	References sequence	Forward primer sequence (5′-3′)	Reverse primer sequence (5′-3′)	Amplicon size (bp)
*GAPDH*	KJ786424.1	AAG​TTC​CAC​GGC​ACA​GTC​AA	CAG​CAT​CGC​CCC​ATT​TGA​TG	109
*Ldh1*	NM_001172363.2	ACA​CTG​GAA​AGC​GGT​TCA​CA	TTC​TGC​CAG​ATC​TGC​CAC​AG	109
*Pkfp*	XM_021065066.1	GGA​GTT​CTG​TGT​CCC​CAT​GG	TGT​CGG​TGA​TGG​TGT​TGA​GG	107
*Pkm2*	CV864390.1	TCA​TTC​AGA​CCC​AGC​AGC​TG	TGG​TAC​AGA​TGA​TGC​CGG​TG	117

### Measurement of ATP Contents

ATP levels in porcine oocytes or their companion cumulus cells (CCs) were assayed using a luciferin–luciferase reaction-based assay kit (Bioluminescent Somatic Cell Assay Kit, Catalog #FL-ASC, St Louis, MO) as previously described (Van Blerkom et al., 1995; Combelles and Albertini, 2003; Xu et al., 2014) with minor changes. Briefly, 20 cumulus-free oocytes or CCs derived from 20 to 30 COCs were placed in microfuge tubes containing 160 and 180 μl water, respectively, and then snap-frozen in liquid nitrogen and stored at −80°C. To determine ATP levels in samples, add 50 μl of each thawed sample or a standard solution of known ATP content to 100 μl ice-cold Cell ATP-Releasing Reagent and incubate for 5 min on ice, then add 100 μl of ice-cold ATP Assay Mix (1:25 dilution in assay mix buffer). The above reaction mixture was then incubated for 10 min at room temperature in the dark for an initial chemiluminescence flash period. Bioluminescence of each sample and ten different standards of known ATP content (0–90 pmol ATP) was measured by a high-sensitivity luminometer. In each measurement, the samples and standards were assayed in three replicates each, and the average value was used to represent the test results. Finally, the ATP content in each sample was calculated according to the 10-point standard curve for each assay. The DNA concentration in the remaining lysed CCs sample was measured and used to normalize the ATP content in each sample. The ATP content in oocytes was presented in pmol/oocyte, while the ATP level in CCs was expressed in pmol/mg DNA.

### Statistical Analysis

Statistical analyses and graphing were performed using GraphPad Prism 7 (GraphPad Software, Inc., San Diego, CA, United States). Each experiment was repeated at least three times, and the specific number of oocytes tested in the experiment is stated in the text. The data are presented as the mean ± standard error of the mean (SEM) of independent experiments and analyzed by one-way ANOVA followed by Tukey multiple comparisons test. Differences at *p* < 0.05 were considered significant.

## Results

### Glycine or Glycine Plus Melatonin Supplementation Significantly Improved the Oocyte Maturation and Subsequent Preimplantation Embryo Development Following COCs Vitrification

**FIGURE 1:**
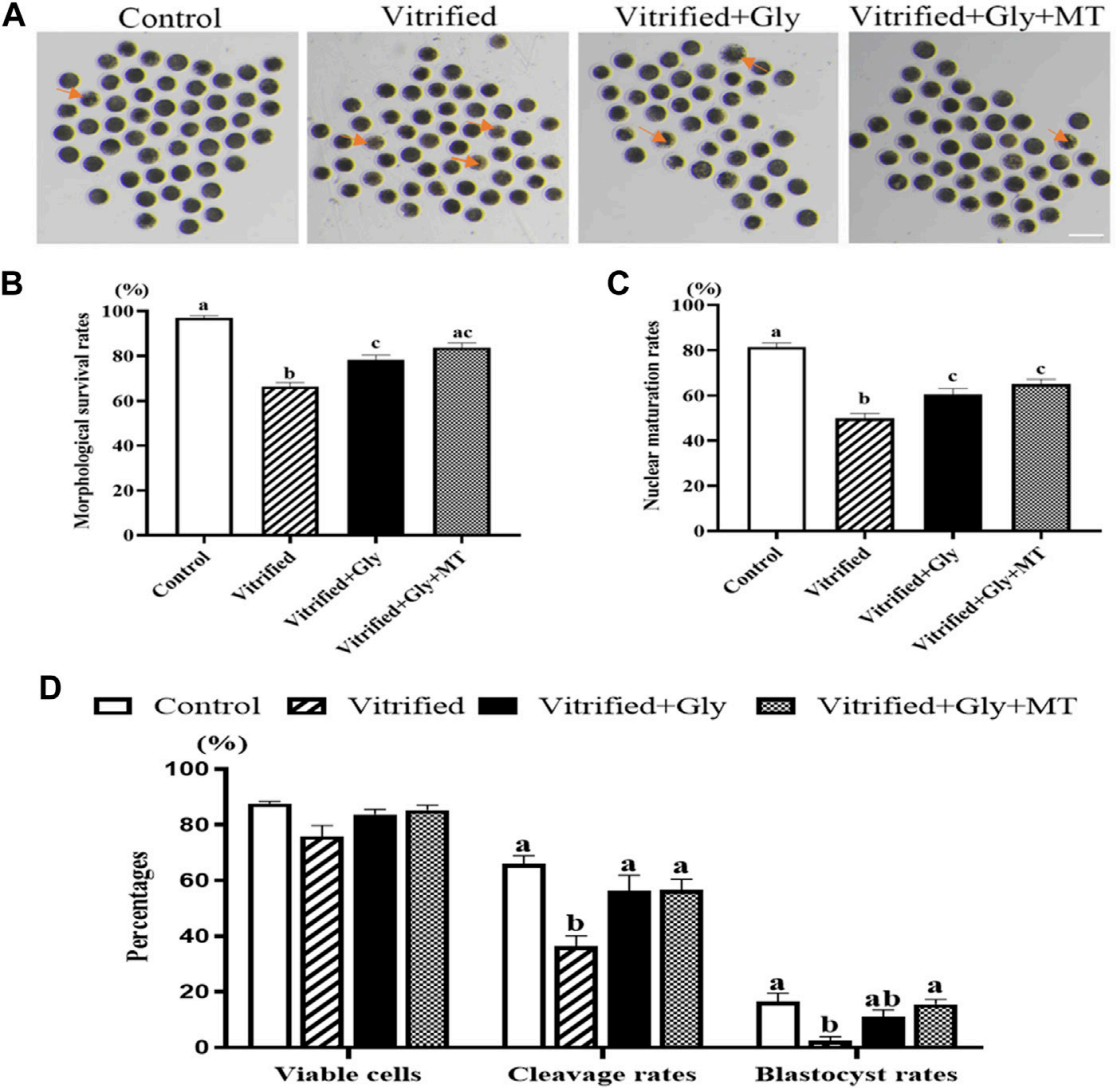
Effects of glycine or glycine plus melatonin supplementation during vitrification, thawing and *in vitro* maturation on oocyte survival, maturation and subsequent preimplantation development. **(A)**: Representative images of oocytes were collected 44 h after maturation *in vitro* to determine whether they were alive or not morphologically in each examined group. The arrows indicate dead cells. Scale bar: 200 μm. **(B)**: The survival rates of oocytes collected from each group. **(C)**: The percentages of oocytes with the first polar body (number of MII oocytes/number of survival oocytes) in each experimental group. **(D)**: The rates of alive oocytes (number of morphologically alive oocytes after IVF/total number of oocytes used for fertilization), cleavage (number of cleaved embryos/number of morphologically viable oocytes after IVF) and blastocyst (number of blastocysts/number of viable oocytes) in each examined group. Control: oocytes were neither vitrified/thawed nor supplemented with glycine or melatonin to assist their maturation; Vitrified: oocytes were sequentially vitrified, thawed and cultured for maturation without glycine or melatonin supplementation; Vitrified + Gly: oocytes were sequentially vitrified, thawed and cultured for maturation with glycine supplementation; Vitrified + Gly + MT: oocytes were sequentially vitrified, thawed and cultured for maturation with glycine plus melatonin supplementation. All experiments were performed in triplicate. Data are shown as mean ± SEM. Different lowercase letters above columns denote statistical difference at *p* < 0.05. The same lowercase letters above columns denote that the data are not significantly different at *p* > 0.05.

Porcine oocytes are extremely sensitive to chilling and easy to die after cryopreservation. To test whether glycine or melatonin increases the viability of porcine oocytes vitrified at the germinal vesicle (GV) stage, the different concentrations of glycine (0, 1, 6, and 10 mM) or melatonin (0, 10^−9^, and 10^−7^ M) were supplemented to the vitrification, thawing and maturation medium, respectively. Oocyte viability and cumulus expansion index assay after maturation culture demonstrated that 6 mM glycine and 10^−9^ M melatonin were the best concentration with the least side effects under our experimental conditions (unpublished data) and were used in the following experiments. To investigate whether the addition of glycine plus melatonin to the vitrification, thawing and maturation media is more effective in enhancing vitrified oocyte survival than glycine alone, porcine COCs were sequentially vitrified, thawed and cultured without any supplementation or supplemented with either 6 mM glycine or 6 mM glycine plus 10^−9^ M melatonin, respectively. In addition, the fresh porcine COCs without vitrification and thawing were used as the control group. As shown in Figure 1A, the viable oocytes in each group had a spherical shape, smooth surface, and evenly granulated cytoplasm with apparent perivitelline space, whereas nonviable oocytes pointed at by an arrow in each group had abnormal shape, degenerated cytoplasm without perivitelline space. Moreover, statistical analysis (Figure 1B) indicated that the survival percentages were dramatically lower in the vitrified/thawed oocytes without any supplementation (Vitrified group, 66.3 ± 1.9%, *n* = 148) compared to those of fresh oocytes without vitrification (control group, 97.0 ± 1.0%, *n* = 158), vitrified/thawed oocytes with 6 mM glycine supplementation (Vitrified + Gly group, 78.3 ± 2.0%, *n* = 138) and vitrified/thawed oocytes with 6 mM glycine plus 10^−9^ M melatonin supplementation (Vitrified + Gly + MT group, 83.4 ± 2.2%, *n* = 138) (*N* = 3, *p* < 0.05). On the other hand, the survival rates were significantly reduced in the Vitrified + Gly group compared to those of the control group (*N* = 3, *p* < 0.05), while there was no obvious difference in the survival rates when the Vitrified + Gly + MT group was compared with either the Vitrified + Gly group or the control group (*N* = 3, *p* > 0.05).

To further examine whether glycine or glycine plus melatonin supplementation promotes the porcine oocyte maturation following vitrification, thawing and maturation of COCs, we compared the percentages of nuclear maturation, manifested by the emission of the first polar body, among the examined groups. Figure 1C demonstrates that the nuclear maturation percentages were dramatically lower in the vitrified group (53.5 ± 1.2%, *n* = 117) compared to those of control group (81.4 ± 1.7%, *n* = 93), Vitrified + Gly group (64.5 ± 0.8%, *n* = 98) and Vitrified + Gly + MT group (65.1 ± 2.1%, *n* = 87) (*N* = 3, *p* < 0.05). Furthermore, the nuclear maturation rates were significantly reduced in both Vitrified + Gly and Vitrified + Gly + MT groups compared to those of control group (*N* = 3, *p* < 0.05), while there was no significant difference between the Vitrified + Gly and the Vitrified + Gly + MT group (*N* = 3, *p* > 0.05).

Removal of excess spermatozoa from the surface of the zona pellucida of porcine oocytes by repeated pipetting after *in vitro* fertilization prevents polyspermy occurrence to some extent. However, it may cause damage to the cytoplasm membrane and cell death. We next compared the effects of glycine or glycine plus melatonin supplementation on the cell death induced by the removal of excess sperms attached to the zona pellucida of oocyte after fertilization and preimplantation embryo development following vitrification, thawing, maturation, and fertilization of porcine oocytes. Our results demonstrated that there was no obvious difference in cell death among the four examined groups (Figure 1D). Moreover, the embryo cleavage rates were significantly decreased in the vitrified group (36.4 ± 2.3%, *n* = 112) compared to those of control group (66.0 ± 2.1%, *n* = 120), Vitrified + Gly group (55.0 ± 3.2%, *n* = 102) and Vitrified + Gly + MT group (55.3 ± 2.8%, *n* = 121) (N = 3, *p* < 0.05). While there was no difference in the embryo cleavage rates of the latter three groups (*N* = 3, *p* > 0.05). Furthermore, the blastocyst percentages were dramatically lower in the vitrified group (2.5 ± 1.2%, *n* = 112) compared to those of control group (15.9 ± 1.7%, *n* = 120) and Vitrified + Gly + MT group (14.2 ± 1.9%, *n* = 121) (*N* = 3, *p* < 0.05). Whereas there was no significant difference in blastocyst percentage between the Vitrified + Gly group (11.0 ± 1.6%, *n* = 102) and the other three groups (*N* = 3, *p* > 0.05).

### The Addition of Glycine or Glycine Plus Melatonin Alleviated the ROS Levels Induced by Freezing and Thawing

**FIGURE 2:**
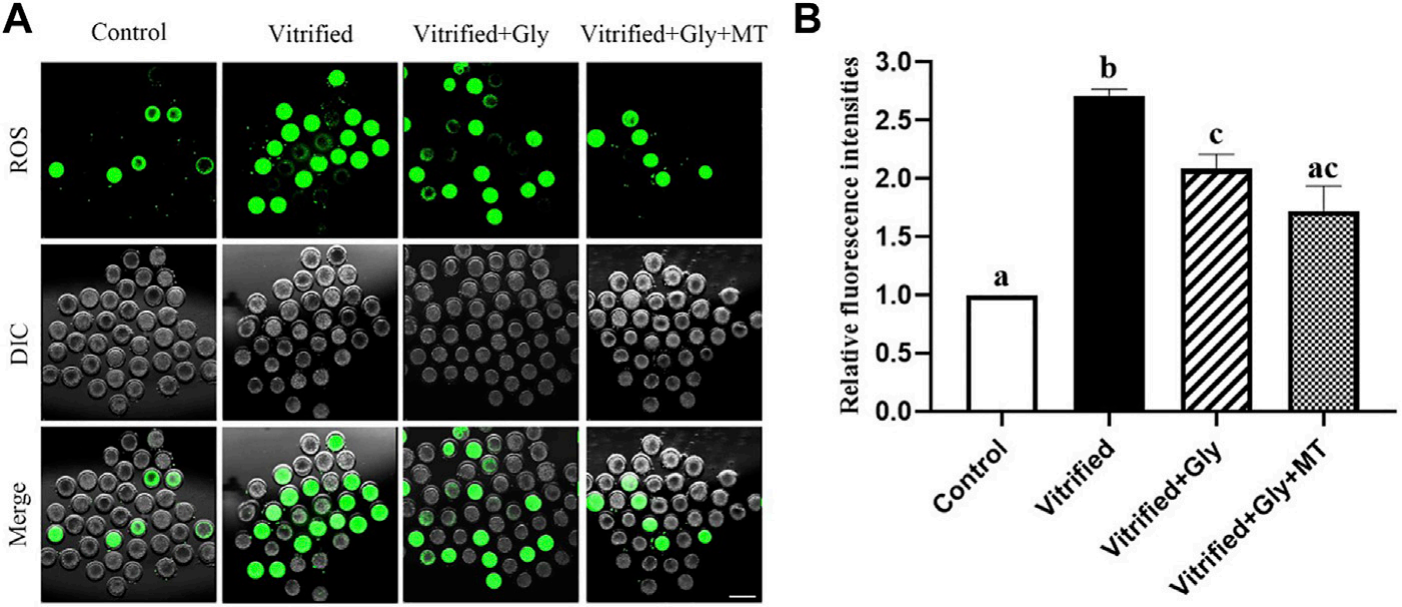
Effects of loading GV oocytes with glycine or glycine plus melatonin during vitrification, thawing and *in vitro* maturation on the intracellular ROS levels of the resultant oocytes. **(A)**: Representative images of ROS staining (green) in oocytes of each experimental group. DIC, differential interference contrast image; Merge, overlapping of green and DIC. Scale bar: 200 μm. **(B)**: The relative fluorescence intensity of ROS staining in oocytes with various treatments. All experiments were performed in triplicate. In each set of experiments, the fluorescence intensity of ROS staining was normalized to the value of oocytes in the control group. Each column presents the mean ± SEM. Different lowercase letters above columns indicate statistical difference at *p* < 0.05. The same lowercase letters above columns denote that the data are not significantly different at *p* > 0.05. Control: oocytes were neither vitrified/thawed nor supplemented with glycine or melatonin to assist their maturation; Vitrified: oocytes were sequentially vitrified, thawed and cultured for maturation without glycine or melatonin supplementation; Vitrified + Gly: oocytes were sequentially vitrified, thawed and cultured for maturation with glycine supplementation; Vitrified + Gly + MT: oocytes were sequentially vitrified, thawed and cultured for maturation with glycine plus melatonin supplementation.

Oxidative stress during vitrification compromises the developmental competency of vitrified oocytes. To explore whether the supplementation of glycine or glycine plus melatonin in the vitrification, thawing and maturation media mitigates oxidative stress induced by vitrification and thawing, we compared the ROS contents in each group after 44 h maturation culture. As shown in Figure 2, the relative fluorescence intensity of the oocytes in the non-vitrified control group (1.0, *n* = 78) was significantly lower than that in both the vitrified group without any supplementation (2.7 ± 0.1, *n* = 58) and the vitrified group supplemented with glycine (2.1 ± 0.1, *n* = 98) (*N* = 3, *p* < 0.05). Whereas, there was no significant difference in ROS intensity between the non-vitrified control group and the vitrified group supplemented with both glycine and melatonin supplementation (1.7 ± 0.2, *n* = 80) (*N* = 3, *p* > 0.05). In addition, the ROS signal in the vitrified oocytes without any supplementation was stronger than that of either glycine-added vitrification group or vitrification group supplemented with glycine plus melatonin (*N* = 3, *p* < 0.05). However, the ROS content of the Vitrified + Gly group was comparable to that of the Vitrified + Gly + MT group (*N* = 3, *p* > 0.05).

### Glycine or Glycine Plus Melatonin Supplementation Attenuated the Damage of COCs Vitrification on the Translocating of Cortical Granules From the Center of the Egg to the Periphery

**FIGURE 3:**
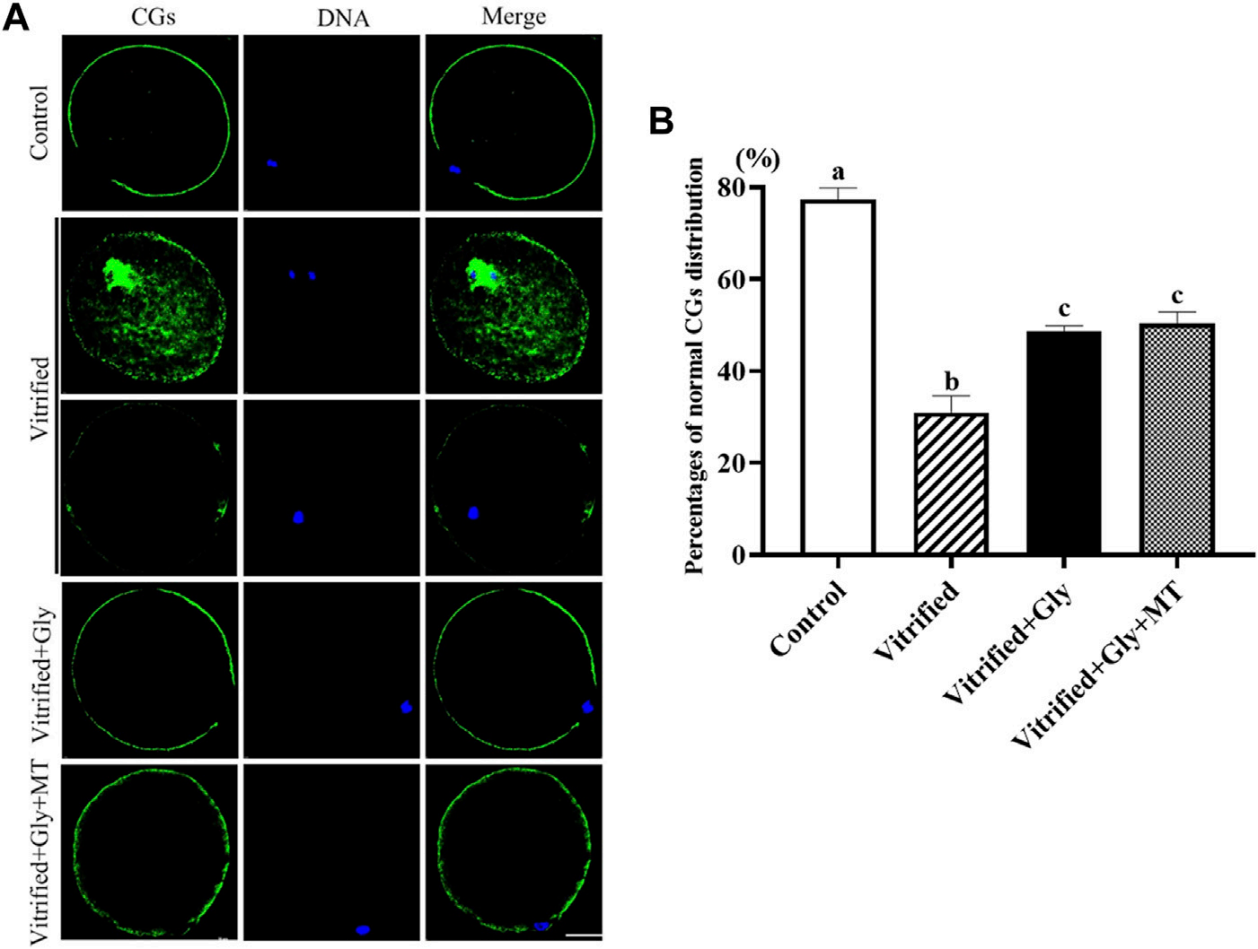
Effects of glycine or glycine plus melatonin addition during vitrification, thawing and *in vitro* maturation on the migration of cortical granules (CGs) in the resultant oocytes. **(A)**: Representative images of the distribution of CGs in oocytes that were collected 44 h after maturation in each examined group. Green, cortical granules (CGs); Blue, chromatin; Merge, overlapping of green and blue. Scale bar: 40 μm. **(B)**: The percentages of oocytes with the normal distribution of GCs in each experimental group. All experiments were performed in triplicate. Data are presented as mean ± SEM of three independent experiments. Different lowercase letters above columns indicate statistical difference at *p* < 0.05. The absence of letters denotes that the data are not significantly different at *p* > 0.05. Control: oocytes were neither vitrified/thawed nor supplemented with glycine or melatonin to assist their maturation; Vitrified: oocytes were sequentially vitrified, thawed and cultured for maturation without glycine or melatonin supplementation; Vitrified + Gly: oocytes were sequentially vitrified, thawed and cultured for maturation with glycine supplementation; Vitrified + Gly + MT: oocytes were sequentially vitrified, thawed and cultured for maturation with glycine plus melatonin supplementation.

The impaired oocyte quality and embryonic development of vitrified oocytes can be attributed in part to defective cortical granule distribution and premature cortical granule exocytosis (Liu, 2011). To investigate whether the supplementation of glycine or glycine plus melatonin in the vitrification, thawing and maturation media mitigates damage to the migration and localization of cortical granules induced by vitrification, we compared the subcellular distribution of cortical granules in each group after 44 h maturation culture. As shown in Figure 3, the vast majority of oocytes (77.3 ± 1.5%, *n* = 89) in the control group had a uniform layer of CGs accumulated in the egg cortex except for the area above the chromosomes known as the “CGs-free zone,” which was recognized as the normal distribution of CGs. Only about a third (31.0 ± 2.1%, *n* = 93) oocytes displayed the normal distribution of CGs, while other oocytes demonstrated either obvious CGs signal in the center of the oocyte or weak, discontinuous layer of CGs in the periphery of the oocyte in the vitrified group. Loading oocytes with either glycine (48.7 ± 0.7%, *n* = 102) or glycine plus melatonin (50.3 ± 1.5%, *n* = 92) during vitrification, thawing and *in vitro* maturation significantly improved CGs transport from the center to the cortex and accumulation in the cortex, and a higher proportion of oocytes with the normal distribution of CGs were found compared with the vitrified group (*N* = 3, *p* < 0.05). However, the ratio of oocytes with the normal distribution of CGs in either Vitrified + Gly or Vitrified + Gly + MT group was dramatically lower than that of the control group (*N* = 3, *p* < 0.05). There was no obvious difference between Vitrified + Gly and Vitrified + Gly + MT groups (*N* = 3, *p* > 0.05).

### Glycine or Glycine Plus Melatonin Treatment Mitigated the Occurrence of Early Oocyte Apoptosis Induced by Vitrification

**FIGURE 4:**
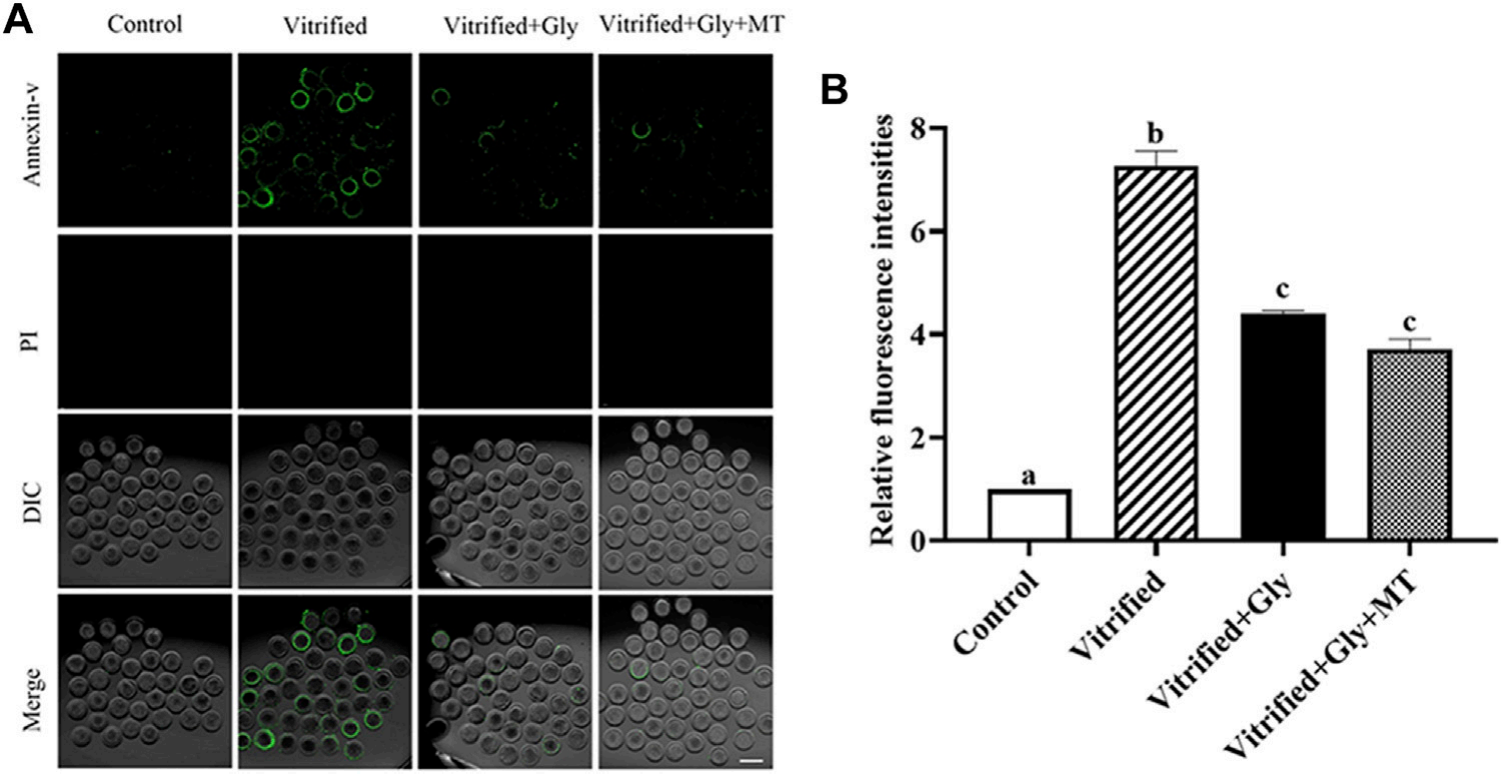
Glycine or glycine plus melatonin supplementation during vitrification, thawing and *in vitro* maturation protected against apoptosis in the subsequent oocytes. **(A)**: Representative images of early apoptosis labeled with Annexin-V in each examined group. Green, Annexin-V staining; PI (propidium iodide), DNA; DIC, differential interference contrast image; Merge, overlapping of green, DNA and DIC. Scale bar: 40 μm. **(B)**: The relative fluorescent intensity of Annexin-V staining in oocytes of each experimental group. In each set of experiments, the fluorescence intensity of Annexin-V staining was normalized to the value of oocytes in the control group. All experiments were performed in triplicate. Data are shown as mean ± SEM. Different lowercase letters above columns indicate statistical difference at *p* < 0.05. The same lowercase letters above columns denote that the data are not significantly different at *p* > 0.05. Control: oocytes were neither vitrified/thawed nor supplemented with glycine or melatonin to assist their maturation; Vitrified: oocytes were sequentially vitrified, thawed and cultured for maturation without glycine or melatonin supplementation; Vitrified + Gly: oocytes were sequentially vitrified, thawed and cultured for maturation with glycine supplementation; Vitrified + Gly + MT: oocytes were sequentially vitrified, thawed and cultured for maturation with glycine plus melatonin supplementation.

Accumulating evidence indicates that vitrification/thawing destroys the membrane of oocytes, induces apoptosis, and impairs the developmental potential of oocytes. To investigate the protective effect of glycine or glycine plus melatonin on oocyte apoptosis during vitrification/thawing and *in vitro* maturation, we detected the Annexin-V staining, a marker of early apoptosis, in oocytes after different treatments. As shown in Figure 4, the Annexin-V signal was rarely observed in oocytes of the control group (1.0, *n* = 89), while the Annexin-V staining was dramatically enhanced in oocytes of the vitrified group (7.3 ± 0.3, *n* = 120) (*N* = 3, *p* < 0.05). On the other hand, the addition of glycine (4.4 ± 0.1, *n* = 102) or glycine plus melatonin (3.7 ± 0.2, *n* = 92) during vitrification, thawing and oocyte maturation significantly attenuated the incidence of apoptosis compared with that of the vitrified group but was still higher than the control group (*N* = 3, *p* < 0.05). There was no obvious difference between glycine and glycine plus melatonin treatments (*N* = 3, *p* > 0.05).

### The Addition of Glycine or Glycine Plus Melatonin Alleviated the Damage of COCs Vitrification on the Distribution of Mitochondria

**FIGURE 5:**
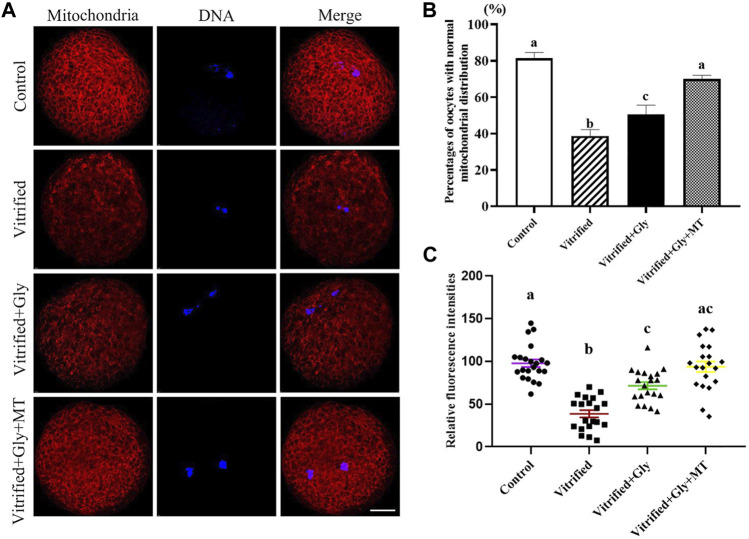
Effects of loading GV oocytes with glycine or glycine plus melatonin during vitrification, thawing and *in vitro* maturation on mitochondrial distribution and activity in the resultant oocytes. **(A)**: Fluorescent staining of functional mitochondrial (red) counterstained with DAPI (blue) in oocytes. Merge, overlapping of red and blue. Scale bar: 20 μm. **(B)**: Percentages of oocytes with the normal mitochondrial distribution. **(C)**: Mitochondrial activity of oocytes with different experimental treatments. Control: oocytes were neither vitrified/thawed nor supplemented with glycine or melatonin to assist their maturation; Vitrified: oocytes were sequentially vitrified, thawed and cultured for maturation without glycine or melatonin supplementation; Vitrified + Gly: oocytes were sequentially vitrified, thawed and cultured for maturation with glycine supplementation; Vitrified + Gly + MT: oocytes were sequentially vitrified, thawed and cultured for maturation with glycine plus melatonin supplementation. All experiments were performed in triplicate. Each column presents the mean ± SEM. Different lowercase letters above columns indicate statistical difference at *p* < 0.05. The same lowercase letters above columns denote that the data are not significantly different at *p* > 0.05.

Mitochondria are the main places where ATP is produced to provide energy to cells. Vitrification normally interrupts the subcellular localization and function of mitochondria in oocytes, which is associated with the developmental competence of oocytes. To verify the effects of glycine or glycine plus melatonin supplementation on the mitochondrial function and distribution during vitrification, thawing and oocyte maturation, we examined the distribution pattern and activity of functional mitochondria in oocytes after various treatments. As shown in Figures 5A,B the majority of oocytes in the control group (81.3 ± 2.6%, *n* = 108) possessed clustered homogeneous mitochondria distribution. In the vitrified group, only part of the oocytes (38.5 ± 2.0%, *n* = 95) had clustered homogeneous mitochondria distribution, while most of the other oocytes presented either fine homogeneous or granulated heterogeneous mitochondria distribution. There were significant differences in mitochondrial distribution patterns between the control group and the vitrified group (*N* = 3, *p* < 0.05). Interestingly, loading oocytes with glycine (50.7 ± 2.8%, *n* = 98) significantly increased the percentages of oocytes with clustered homogeneous mitochondria distribution compared with the vitrified group. Moreover, loading oocytes with both glycine and melatonin (70.0 ± 1.6%, *n* = 102) could further improve the rates of oocytes with clustered homogeneous mitochondria distribution compared with either the vitrified group or the Vitrified + Gly group (*N* = 3, *p* < 0.05). There was no significant difference in the mitochondrial distribution pattern between the Vitrified + Gly + MT group and the control group (*N* = 3, *p* > 0.05). Additionally, mitochondrial activity as measured by fluorescence intensity was closely related to the type of mitochondrial distribution. The lowest fluorescence intensity/oocyte (38.6 ± 4.3, *n* = 20) was found in the vitrified group. The intensity of fluorescence increased in vitrified oocytes with either glycine (71.5 ± 4.3, *n* = 20) or glycine plus melatonin (93.7 ± 6.2, *n* = 20) treatment, and reached the top level in the control oocytes (97.8 ± 4.4, *n* = 20) (Figure 5C). Statistical analysis showed that the differences in the mitochondrial activity of oocytes in different experimental groups were consistent with the type of mitochondrial distribution type, except that there was no significant difference in mitochondrial activity between the Vitrified + Gly group and the Vitrified + Gly + MT group (*N* = 3, *p* > 0.05) (Figures 5B,C).

### Glycine or Glycine Plus Melatonin Addition Promoted Glycolytic Gene Expression in CCs

**FIGURE 6:**
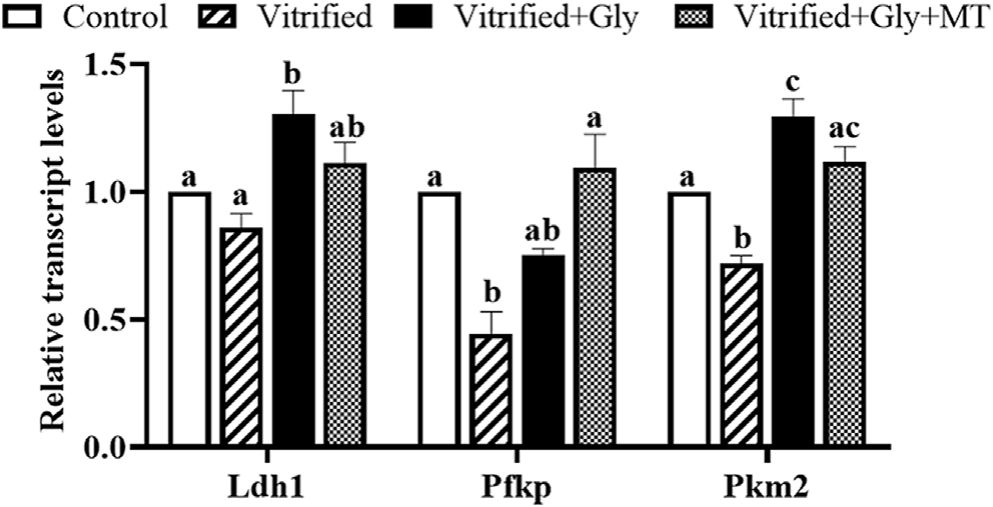
Glycine or glycine plus melatonin supplementation during vitrification/thawing and *in vitro* maturation increased the relative transcript levels of glycolytic genes in the CCs. In each set of experiments, the transcript levels of the glycolytic genes of each group were normalized to the value of CCs in the control group. Control: oocytes were neither vitrified/thawed nor supplemented with glycine or melatonin to assist their maturation; Vitrified: oocytes were sequentially vitrified, thawed and cultured for maturation without glycine or melatonin supplementation; Vitrified + Gly: oocytes were sequentially vitrified, thawed and cultured for maturation with glycine supplementation; Vitrified + Gly + MT: oocytes were sequentially vitrified, thawed and cultured for maturation with glycine plus melatonin supplementation. Each column indicates the mean ± SEM. Different lowercase letters above columns indicate statistical difference at *p* < 0.05. The same lowercase letters above columns denote that the data are not significantly different at *p* > 0.05.

ATP levels in oocytes are closely related to glycolytic gene expression levels in their companion CCs both *in vivo* and *in vitro*. To evaluate whether the supplementation of glycine or glycine plus melatonin in the vitrification, thawing and maturation media protect the bi-directional communication between porcine oocytes and their companion CCs, thereby maintaining the levels of glycolytic gene expression in CCs from cryoinjury, we compared the glycolytic gene transcript levels of *Ldh1*, *Pkm2*, and *Pfkp* in the CCs isolated from each experimental group. *Ldh1 e*ncodes lactate dehydrogenase, which mediates bidirectional conversion between pyruvate and lactate, thereby regulating their homeostasis, while *Pkm2* (pyruvate kinase) is required for the conversion of phosphoenolpyruvate to pyruvate. *Pfkp* (phosphofructokinase) is the rate-limiting enzyme of the pentose phosphate pathway that regulates glucose metabolism. Our data demonstrated that the transcript levels of *Pfkp* and *Pkm2* were dramatically decreased in the CCs of the vitrified group compared with the control group (*N* = 3, *p* < 0.05), whereas those of the *Ldh1* were comparable between the vitrified group and control group (*N* = 3, *p* > 0.05) (Figure 6). Glycine treatment dramatically improved the transcript levels of *Pkm2* and *Ldh1* in CCs in the Vitrified + Gly group compared with either control or vitrified group (*N* = 3, *p* < 0.05). Whereas glycine plus melatonin treatment significantly increased the transcript levels of *Pkm2* in CCs compared to those in the vitrified group (*N* = 3, *p* < 0.05). However, there was no obvious difference between the Vitrified + Gly + MT group and the control group or between the Vitrified + Gly + MT group and the Vitrified + Gly group in the transcript levels of *Pkm2* and *Ldh1* in CCs (*N* = 3, *p* > 0.05). On the other hand, glycine or glycine plus melatonin treatment significantly boosted the transcript levels of *Pfkp* in the CCs (*N* = 3, *p* < 0.05) to comparable levels in the control group (*N* = 3, *p* > 0.05) (Figure 6).

### Glycine or Glycine Plus Melatonin Treatment Increased ATP Contents in Vitrified MII Oocyte and Its Companion Cumulus Cells

**FIGURE 7:**
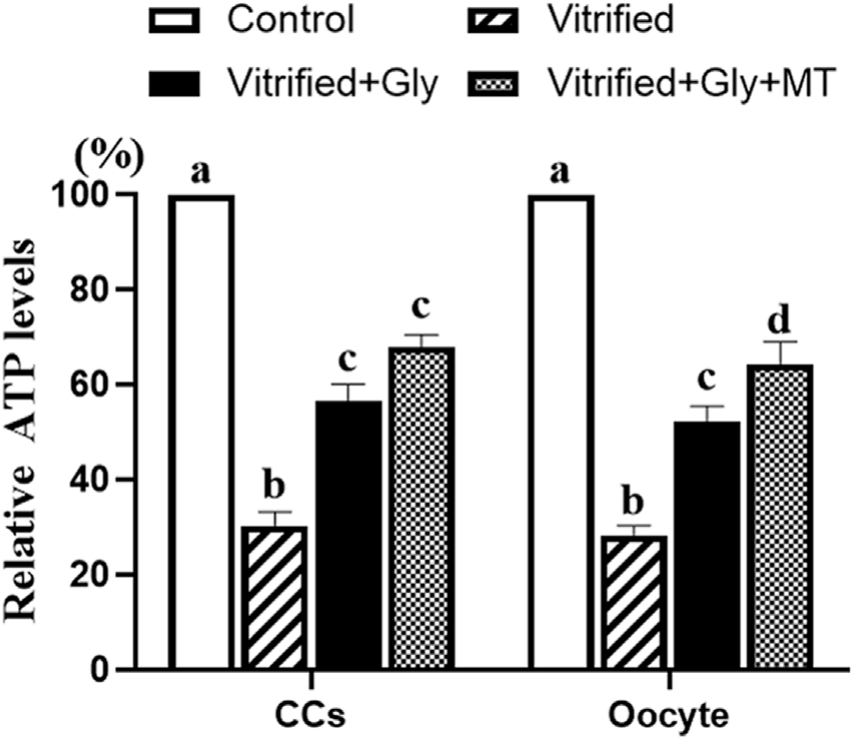
Effects of loading GV oocytes with glycine or glycine plus melatonin during vitrification, thawing and *in vitro* maturation on the relative ATP levels in oocytes and CCs. In each set of experiments, the ATP contents of differently treated oocytes or CCs were normalized against the value of oocytes or CCs in the control group, respectively. Control: oocytes were neither vitrified/thawed nor supplemented with glycine or melatonin to assist their maturation; Vitrified: oocytes were sequentially vitrified, thawed and cultured for maturation without glycine or melatonin supplementation; Vitrified + Gly: oocytes were sequentially vitrified, thawed and cultured for maturation with glycine supplementation; Vitrified + Gly + MT: oocytes were sequentially vitrified, thawed and cultured for maturation with glycine plus melatonin supplementation. All experiments were performed in triplicate. Each column indicates the mean ± SEM. Different lowercase letters above columns indicate statistical difference at *p* < 0.05. The same lowercase letters above columns denote that the data are not significantly different at *p* > 0.05.

Dysfunction of oocyte mitochondrial and deficiency of glycolytic gene expression in CCs are negatively related to the ATP contents of oocytes. To further test whether the addition of glycine or glycine plus melatonin in the vitrification, thawing and maturation media alleviates damage to the ATP production in oocytes and CCs induced by vitrification of COCs, we detected the ATP levels of oocyte and CCs from each experimental group. Our data demonstrated that the vitrification of COCs with or without glycine or glycine plus melatonin supplementation dramatically lessened the ATP contents in both oocytes and CCs compared to those in the control group (*N* = 3, *p* < 0.05). However, glycine or glycine plus melatonin treatment significantly increased the ATP levels in both oocytes and CCs compared with the vitrified group (*N* = 3, *p* < 0.05). In addition, ATP production in CCs was not significantly different between the Vitrified + Gly group and the Vitrified + Gly + MT group (N = 3, *p* > 0.05), while ATP levels in oocytes of the Vitrified + Gly + MT group were higher than these of the Vitrified + Gly group (N = 3, *p* < 0.05) (Figure 7).

## Discussion

Our current results demonstrate that glycine or glycine plus melatonin supplementation during vitrification, thawing, and oocyte maturation improves the quality of oocyte meiotic maturation and subsequent embryonic development after vitrification of porcine COCs. We predicted that reducing the detrimental osmotic injury during dehydration and rehydration of the vitrification process by optimizing intracellular osmotic support with organic osmolytes can improve the developmental competency of vitrified porcine oocytes through protecting organelles and bi-directional communication between oocyte and its companion CCs from irreversible damage and lowering the oxidative stress. We found that glycine treatment of vitrified oocytes restored the ROS levels, the mitochondrial physiology, the migration of cortical granules from the center of the cell to the periphery, ATP contents and glycolytic gene expression in its companion CCs, inhibited apoptotic occurrence, thus resuming blastocyst development. Moreover, we also evaluated the effects of glycine plus melatonin treatment of vitrified oocytes, which is supposed to alleviate the osmotic and oxidative stresses simultaneously, on organelle physiology, communication between oocyte and its neighboring CCs, and developmental competency of the blastocyst. Our data indicated that the beneficial effects of loading oocytes with either glycine or glycine plus melatonin were comparable, but the latter was more likely to increase ATP contents in oocytes and normal mitochondrial distribution in oocytes than the former. Taken together, the osmotic stress and oxidative stress during vitrification and thawing of mammalian oocytes may cause cellular damage mainly through similar molecular mechanisms, at least to some extent, and reducing the damage of one can also alleviate the damage of the other. On the other hand, oxidative stress also exerts mild biological function independently from osmotic stress because amelioration of both osmotic and oxidative stresses during vitrification, thawing and maturation systems has a further ability to minimize the cryodamage in the porcine oocytes compared with lowering osmotic stress alone.

Glycine is the smallest amino acid and consists of a single carbon molecule linked to an amino acid and a carboxyl group (Roth, 2007). It has been reported that glycine is the highest abundant amino acid in the sow oviduct and uterine fluids (Iritani et al., 1974), which suggest that glycine might play important roles in porcine oocyte meiosis and early embryo development *in vivo* and may be an important factor to be considered for the improvement of oocyte maturation and preimplantation embryo culture *in vitro*. Although the role of glycine in mammalian oocyte meiosis and preimplantation embryo development has not been elucidated, there is increasing evidence that it can act as an organic osmolyte in mouse (Dawson et al., 1998; Tartia et al., 2009) and bovine (Elhassan et al., 2001) embryo. While other studies suggest that besides glutamate and cysteine, glycine is another tripeptide component of glutathione (GSH) (Dringen, 2000) and plays an important role in protection from oxidative stress during meiotic division and early embryo development of porcine oocytes (Li et al., 2018). GSH is the most abundant intracellular antioxidant thiol and is central to redox defense during oxidative stress (Biswas and Rahman, 2009). However, the intracellular level of cysteine is much lower than those of the other two substrates, glutamate and glycine, and cysteine is the rate-limiting substrate for GSH synthesis (Iritani et al., 1974; Dringen et al., 1999a; Dringen et al., 1999b). Therefore, we suppose that glycine treatment enhances the developmental potential of porcine oocytes and preimplantation embryos not through GSH production. Our previous (Cao et al., 2016) and current studies demonstrate that glycine is also an efficient organic cryoprotectant for improving the vitrification efficiency of mouse and porcine oocytes, respectively. Whether glycine or other organic osmolytes can be used as a cryoprotectant for oocytes or preimplantation embryos of other animals remains to be elucidated.

Melatonin is another endogenous antioxidant and metabolic regulator found in porcine follicular fluid (Shi et al., 2009; Jin et al., 2017; He et al., 2018; Liu et al., 2018). Its concentration in porcine follicular fluid is approximately 10^−11^ M, which is negatively correlated with follicle size, with a trend of smaller follicles and higher melatonin content (Shi et al., 2009). Melatonin promotes *in vitro* maturation and parthenogenetic development of porcine oocytes in a dose-dependent manner, but high concentrations have negative effects (Shi et al., 2009; Jin et al., 2017), in two ways (Jiang et al., 2021). It either directly chelates oxygen and nitrogen reactive species as well as mobilizes the antioxidant enzyme in cells (Amaral and Cipolla-Neto, 2018) or binds to its receptors (Reiter et al., 2014) to decrease downstream molecules, such as cAMP and cGMP, as well as increase PLC (Jockers et al., 2016), to mitigate oxidative stress in the female reproductive system. In the present study, 10^−9^ M melatonin was used to explore its impacts on the vitrification efficiency of porcine COCs according to the results of three different concentrations tested. Although this concentration is also the optimal concentration for improving the *in vitro* maturation quality of porcine oocytes in previous studies, more studies related to the concentration gradient of melatonin are needed to screen the optimal concentration for improving the vitrification efficiency of porcine oocytes. In addition, RT-PCR analysis indicates that the transcripts of melatonin receptor 1 (MT1) are present in porcine cumulus and granulosa cells but not in porcine oocytes (Kang et al., 2009). Whereas, immunofluorescence assay of porcine COCs demonstrates that melatonin receptor 2 (MT2) is expressed in both oocytes and CCs. The effect of melatonin on enhancing blastocyst formation rate and total blastocyst cell number after parthenogenesis can be eliminated by specific MT2 inhibitors (Lee et al., 2018). Another study also proves that melatonin can boost the synthesis of estradiol in porcine granulosa cells (Liu et al., 2018). These results suggest that melatonin may promote porcine oocyte meiotic maturation and subsequent embryonic development through its receptor and communication with its companion somatic cells to exert its biological functions. Consistent with these findings, the addition of glycine plus melatonin was more potent in increasing the ATP content of vitrified porcine CCs than glycine addition alone, although there was no statistical difference between them in the present results. Moreover, melatonin treatment during porcine oocyte maturation *in vitro* increases the lipid droplets (LDs) accumulation (Jin et al., 2017; He et al., 2018) and the transcript levels of genes involved in lipogenesis, lipolysis and fatty acid beta-oxidation (Jin et al., 2017). This may explain why the supplementation of glycine plus melatonin was more effective in increasing the ATP contents in the vitrified porcine oocytes than the glycine supplementation alone. Due to the limitations of experimental technology and manpower, the effects of the melatonin addition on vitrification of porcine COCs were not separately examined in this study, so it is impossible to compare the effects of glycine or melatonin addition on the developmental competency of vitrified porcine COCs. More detailed and in-depth experiments are required to investigate the role of melatonin supplementation or the combination of melatonin with oocyte- or preimplantation-embryo-specific osmolyte(s), including glycine, in modulating vitrification of porcine COCs and other lipid-rich oocytes and their regulatory mechanisms.

In conclusion, either glycine or glycine plus melatonin supplementation in the systems of vitrification/thawing and subsequent oocyte maturation of porcine COCs can improve the developmental competency of vitrified porcine oocytes. While loading vitrified porcine oocytes with both glycine and melatonin can further reduce the cryoinjury of oocytes compared with loading oocytes with glycine alone.

## Data Availability

The original contributions presented in the study are included in the article/supplementary material, further inquiries can be directed to the corresponding author.
